# A novel oxidative stress-related gene signature as an indicator of prognosis and immunotherapy responses in HNSCC

**DOI:** 10.18632/aging.205323

**Published:** 2023-12-28

**Authors:** Zhuoqi Li, Chunning Zheng, Hongtao Liu, Jiling Lv, Yuanyuan Wang, Kai Zhang, Shuai Kong, Feng Chen, Yongmei Kong, Xiaowei Yang, Yuxia Cheng, Zhensong Yang, Chi Zhang, Yuan Tian

**Affiliations:** 1 Department of Otolaryngology-Head and Neck Surgery, Shandong Provincial ENT Hospital, Shandong University, Jinan, Shandong 250299, P.R. China; 2 Radiotherapy Department, Shandong Second Provincial General Hospital, Shandong University, Jinan, Shandong 250299, P.R. China; 3 Department of Gastrointestinal Surgery, Shandong Provincial Hospital Affiliated to Shandong First Medical University, Jinan, Shandong 250021, P.R. China; 4 Department of Pathology, The First Affiliated Hospital of Shandong First Medical University and Shandong Provincial Qianfoshan Hospital, Shandong Medicine and Health Key Laboratory of Clinical Pathology, Shandong Lung Cancer Institute, Shandong Institute of Nephrology, Jinan, Shandong 250014, P.R. China; 5 Department of Respiratory and Critical Care Medicine, Shandong Second Provincial General Hospital, Jinan, Shandong 250299, P.R. China; 6 Department of Oncology, The Second Affiliated Hospital of Shandong University of Traditional Chinese Medicine, Jinan, Shandong 250299, P.R. China; 7 Generalsurgery Department, Wenshang County People’s Hospital, Wenshang, Shandong 272500, P.R. China; 8 Department of Thoracic Surgery, Shandong Cancer Hospital and Institute, Shandong First Medical University and Shandong Academy of Medical Sciences, Jinan, Shandong 250117, P.R. China; 9 Department of Hepatobiliary Intervention, Beijing Tsinghua Changgung Hospital, School of Clinical Medicine, Tsinghua University, Beijing 102218, P.R. China; 10 Department of Gastrointestinal Surgery, Yantai Yuhuangding Hospital, Qingdao University, Yantai, Shandong 264000, P.R. China; 11 Department of Cardiology, The Second Hospital, Cheeloo College of Medicine, Shandong University, Jinan, Shandong 250033, P.R. China

**Keywords:** oxidative stress, HNSCC, OSRS, scRNA-seq, RNA-seq, prognosis

## Abstract

Purpose: To identify molecular subtypes of oxidative stress-related genes in head and neck squamous cell carcinoma (HNSCC) and to construct a scoring model of oxidative stress-related genes.

Methods: R language based scRNA-seq and bulk RNA-seq analyses were used to identify molecular isoforms of oxidative stress-related genes in HNSCC. An oxidative stress-related gene scoring (OSRS) model was constructed, which were verified through online data and immunohistochemical staining of clinical samples.

Results: Using TCGA-HNSCC datasets, nine predictive genes for overall patient survival, rarely reported in previous similar studies, were screened. AREG and CES1 were identified as prognostic risk factors. CSTA, FDCSP, JCHAIN, IFFO2, PGLYRP4, SPOCK2 and SPINK6 were identified as prognostic factors. Collectively, all genes formed a prognostic risk signature model for oxidative stress in HNSCC, which were validated in GSE41613, GSE103322 and PRJEB23709 datasets. Immunohistochemical staining of SPINK6 in nasopharyngeal cancer samples validated the gene panel. Subsequent analysis indicated that subgroups of the oxidative stress prognostic signature played important roles during cellular communication, the immune microenvironment, the differential activation of transcription factors, oxidative stress and immunotherapeutic responses.

Conclusions: The risk model might predict HNSCC prognosis and immunotherapeutic responses.

## INTRODUCTION

Head and neck squamous cell carcinoma (HNSCC) are the most common malignancy in the head and neck, developing from the mucosal epithelium in the oral cavity, pharynx and larynx [1]. GLOBOCAN cancer statistics estimated that 467,125 HNSCC-related deaths occurred in 2020 alone [2]. HNSCC treatment included surgery, radiation, chemotherapy and immunotherapy [1, 3]. Local recurrence and metastasis, particularly for advanced stages were common, with 5-year overall survival (OS) rates of less than 50% [3, 4]. Understanding the molecular mechanisms of HNSCC, a novel and reliable model for prognosis and treatment responses predicting was needed.

The composition of the tumor microenvironment (TME) was complex and diverse [5, 6]. The TME could be either tumor-suppressive or supporting dependent on the stage of tumor progression and the associated organ [7, 8]. Components of the TME could suppress antitumoral responses, including tumor-associated macrophages (TAM), MDSCs, Tregs, PD-1 and PD-L1. The TME also affected therapeutic responses, particularly to ICI immunotherapy [5–8]. In HNSCC, oxidative stress activated NF-κB and STAT3 in CAFs, resulting in CCL2 expression and a cytokine-rich TME increased [9]. Cigarette smoke increased oxidative stress in the TME of HNSCC and induces the expression of MCT4 in fibroblasts, promoting CCL2 expression and macrophage migration [10]. Reversing pro-tumoral M2 to anti-tumoral M1 macrophages could be achieved through targeting oxidative stress-related factors, as M1 macrophages presented significantly higher ROS levels than M2 in HNSCC TME [11]. Therefore, we speculate that there may be a certain correlation between oxidative stress related genes and TME that needs to be revealed.

To reveal the impact of oxidative stress related genes on TME and patient prognosis, this study was designed and put into practice. Relevant indicators would be validated using online data and clinical sample data.

## MATERIALS AND METHODS

### Data collection and processing

The analysis data packages related to the R language used in this study were downloaded from online website (https://cloud.r-project.org). FPKM (fragments per kilobase of exon per million fragments mapped) expression profiles of TCGA-HNSCC were downloaded using the R package “TCGAbiolinks”. Overall survival (OS) and clinical characteristics (including age, stage and gender) were also obtained (Table 1) [12]. In total, 494 tumor samples were collected and analyzed. Expression profiles and clinical information of the GSE41613 dataset were downloaded from the GEO database (https://www.ncbi.nlm.nih.gov/geo/). A total of 97 tumor samples were assessed for expression and survival information as validation for the cohort (Table 1). GSE103322 involving single-cell datasets were downloaded from the GEO database (https://www.ncbi.nlm.nih.gov/geo/), which contained 18 primary tumor samples and a single-cell transcriptome of 5902 cells after initial quality control. A total of 2205 cells were classified as malignancy.

**Table 1:** The clinical information of the samples in TCGA and GSE41613.

**Characteristic**	**Value**	**TCGA-HNSCC**	**GSE41613**
		**sample_num**	**sample_num**
Age, *n* (%)	≥60	216	47
	<60	278	50
Gender, *n* (%)	Female	132	–
	Male	362	–
Pathological Stage, *n* (%)	I/II	93	41
	III/IV	334	56
	unknown	67	–

The PRJEB23709 relating to immunotherapy cohort was downloaded from the BioProject database and used to assess the predictive efficacy of the signatures for immunotherapy [13]. Seventy-seven oxidative stress-related genes were retrieved from the website Harmonizome (https://maayanlab.cloud/Harmonizome/dataset/Biocarta+Pathways), of which 74 were expressed in the training cohort. Subsequent analyses were performed based on those genes.

### Consensus clustering analysis

Unsupervised clustering analysis was applied to identify different oxidative stress gene expression patterns using the R package “ConsensusClusterPlus”. The distance used for clustering was Euclidean. The clustering method was “km”. A total of 1000 replications were performed to ensure the stability of clustering.

Using the R package “survminer” and “survival”, survival curves for prognostic analysis were generated using the Kaplan-Meier method. Log-rank tests were used to determine significant differences and to identify the correlation between expression patterns and OS.

### DEGs

The R package “limma” was used to identify DEGs. Genes with different multiplicities |log2FC|≥1 (difference multiplicity greater than or equal to 2) and FDR <0.05 were screened and included.

### Prognostic signatures

One-way Cox regression analysis was used to determine the hazard ratio (HR) and prognostic significance of the DEGs. Genes with *p* < 0.05 were screened as the prognostic genes. Key prognostic factors were further screened through LASSO regression analysis using the R package “glmnet”. A risk scoring model for patient survival was established through weighting the expression of each key prognostic factor with the LASSO regression coefficient (“χi” represents gene expression level and “βi” represents LASSO regression coefficient) [14, 15]:


Score=∑βi × Xi

Samples were divided into high- and low- score groups according to the median values. Survival curves for prognostic analyses were generated using the Kaplan-Meier method. Significant differences were calculated using log-rank tests to reveal the correlations between samples and OS. Reliability was evaluated using the receiver operating characteristic (ROC) curve. The area under the curve (AUC) was visualized using the R package “timeROC”. Univariate and Multivariate Cox analyses were performed to explore the independent prognostic values of the oxidative stress-related Score (OSRS).

### GSVA (Gene set variation analysis) and functional enrichment analysis

The R package “clusterProfiler” was used to perform GO and KEGG pathway enrichment analyses (parameters pvalueCutoff = 0.05, pAdjustMethod = “BH”). The R package “GSVA” was used to annotate the potential functions of key genes. GSVA is a non-parametric, unsupervised method primarily used to estimate alterations in pathways and biological processes in samples. Gene sets were downloaded from three sub libraries of HALLMARK, KEGG, and GOBP in the MSigDB database for GSVA analyses.

### Tumor immune microenvironment assessment

Immune cell infiltration was compared in different oxidative stress-related groups using the “Wilcoxon” test. The ssGSEA (single-sample gene-set enrichment analysis) was used to estimate the relative abundance of each cell infiltrating in the TME. Gene sets were used to evaluate the infiltrating fraction of each immune cell type in the TME, which contained 28 human immune cell types, including activated CD8^+^ T cells, dendritic cells and macrophages [16]. Enrichment analyses calculated using the ssGSEA were used to indicate the relative abundance of TME-infiltrating cells in each sample. CIBERSORT combined with LM22 feature matrix were used to estimate the proportion of immune cell types in each sample. The sum of the proportions of all estimated immune cell types for each sample was equal to 1.

The proportion of 64 immune cells were calculated based on the “xCell” method in the R package “IOBR”. Immune, stromal, and purity scores were calculated for each tumor sample using the “ESTIMATE” algorithm. The “Wilcoxon” test was used for inter-group comparisons.

### Drug sensitivity prediction

IC50 values in the training cohort were evaluated using the calcPhenotype algorithm of R package “oncoPredict” based on the GDSC (Genomics of Drug Sensitivity in Cancer) (https://www.cancerrxgene.org/) and CTRP (Cancer Therapeutics Response Portal) (https://portals.broadinstitute.org/ctrp/) cancer genomics drug sensitivity database. Spearman’s correlation analyses were performed between the OSRS and IC50 values to determine the correlation between drug sensitivity and oxidative stress-related signatures. Differences were compared between high- and low- scoring groups.

### Quality control for the single-cell transcriptome data

A total of 18 primary tumor samples and 5902 single-cell transcriptome analyses were retained from the following quality control. Clear annotations of all cell types were provided. Data were normalized using the “NormalizeData” function. The top 3000 highly variable genes were identified using the “FindVariableFeatures” function. Batch correction was performed using the R package “Harmony”. Scale transformations and principal component analyses were performed to reduce dimensions. The top 50 principal components were selected for downstream analyses.

### Delineating subgroups for malignant cells

Malignant epithelial cells were extracted. Following the normalization and uniformization, the top 3000 highly variable genes were obtained. The top 50 principal components were selected. Resolution was set at 0.05. Clustering analyses were performed to identify malignant subgroups. Differentially characterized genes amongst screened subgroups were identified using “FindAllMarkers” (avg_log2fc > 0.25, *p*_val_adj < 0.05).

### Trajectory and cell communication analyses

The R package “monocle2” was used for trajectory and pseudotime analyses of malignant cells. The transformations of malignant tumor cells were mapped according to states. Communication analyses between immune and malignant tumor cells were performed using the R package “CellChat”.

### Construction of the transcription factor regulatory network

The motif annotations of human transcription factors and motifs corresponding to gene ranks were downloaded from the “RcisTarget” database (https://resources.aertslab.org/cistarget/). Lists of human transcription factors were downloaded from the following website (https://github.com/aertslab/pySCENIC/tree/master/resources). Transcription factor regulatory networks were constructed based on the R package “SCENIC”. The “AUCell” algorithm was used to calculate the activity of each transcription Factor. Regulatory modules (regulon modules) were identified based on the Connection Specificity Index (CSI). Overall activity scores of regulatory modules were defined as the mean value of all TF activities.

### Sample collection and immunohistochemistry

Nasopharyngeal squamous cell carcinoma samples were collected from the First Affiliated Hospital of Shandong First Medical University from 2013 to 2021 (Table 2). Written informed consents were provided by all participants. Tumor tissues were obtained from biopsy excision, formalin fixed and paraffin embedded (FFPE) for histological evaluation. After paraffin wax removal and rehydration, the sections were placed in blocking buffer (0.5% Triton X-100 and 5% natural goat serum, 0.1 M PBS) for 1 hour at room temperature. Then antigen retrieval was performed with EDTA (pH = 8.0) for 30 minutes. Sections were then placed on the primary antibody (rabbit anti-human SPINK6 polyclonal antibody, 1:400, CSB-PA744263LA01HU, Cusabio Technology LLC, USA) at 1 hour at room temperature. After 3 × 3-minute 0.1 M PBS washes, the sections were incubated in biotinylated secondary antibody at room temperature for 30 min, followed by subsequent washes (3 × 3 min in 0.1 M PBS). After immunostaining, sections were visualized using an HRP conjugated SP system using Leica Bond^™^ System according to the manufacturer’s protocol. Slides were examined by two experienced pathologists independently according to WHO criteria.

**Table 2:** The characteristics of patients with nasopharyngeal squamous cell carcinoma.

**Characteristics**	**Overall**
**Gender, *n* (%)**	
Female	3 (30%)
Male	7 (70%)
Age, mean ± SD	65.2 ± 12.917
**Smoker, *n* (%)**	
No	6 (60%)
Yes	4 (40%)
**Alcohol history, *n* (%)**	
No	6 (60%)
Yes	4 (40%)
**SPINK6 IHC Score, *n* (%)**	
60	1 (10%)
140	1 (10%)
160	5 (50%)
180	1 (10%)
120	1 (10%)
170	1 (10%)

### Statistical analysis

Analyses were performed using R software (version 4.1.2). Individual group analyses were performed using a Wilcoxon rank sum test. A Kruskal-Wallis test was used to compare differences between multiple groups.

OS curves were determined using Kaplan–Meier analysis. Univariate and Multivariate Cox proportional hazard regression models were constructed based on the analysis of prognostic data. Nomogram and Calibration models were further constructed. For plot presentation, where ns indicates *p* > 0.05, ^*^p < 0.05, ^**^stands for *p* < 0.01, ^***^means *p* < 0.001, and ^****^indicates *p* < 0.0001.

## RESULTS

### Sample subgroups by consensus clustering analysis and constructing an oxidative stress-related scoring model

#### Consensus clustering of oxidative stress genes to identify sample subgroups

**Figure 1:**
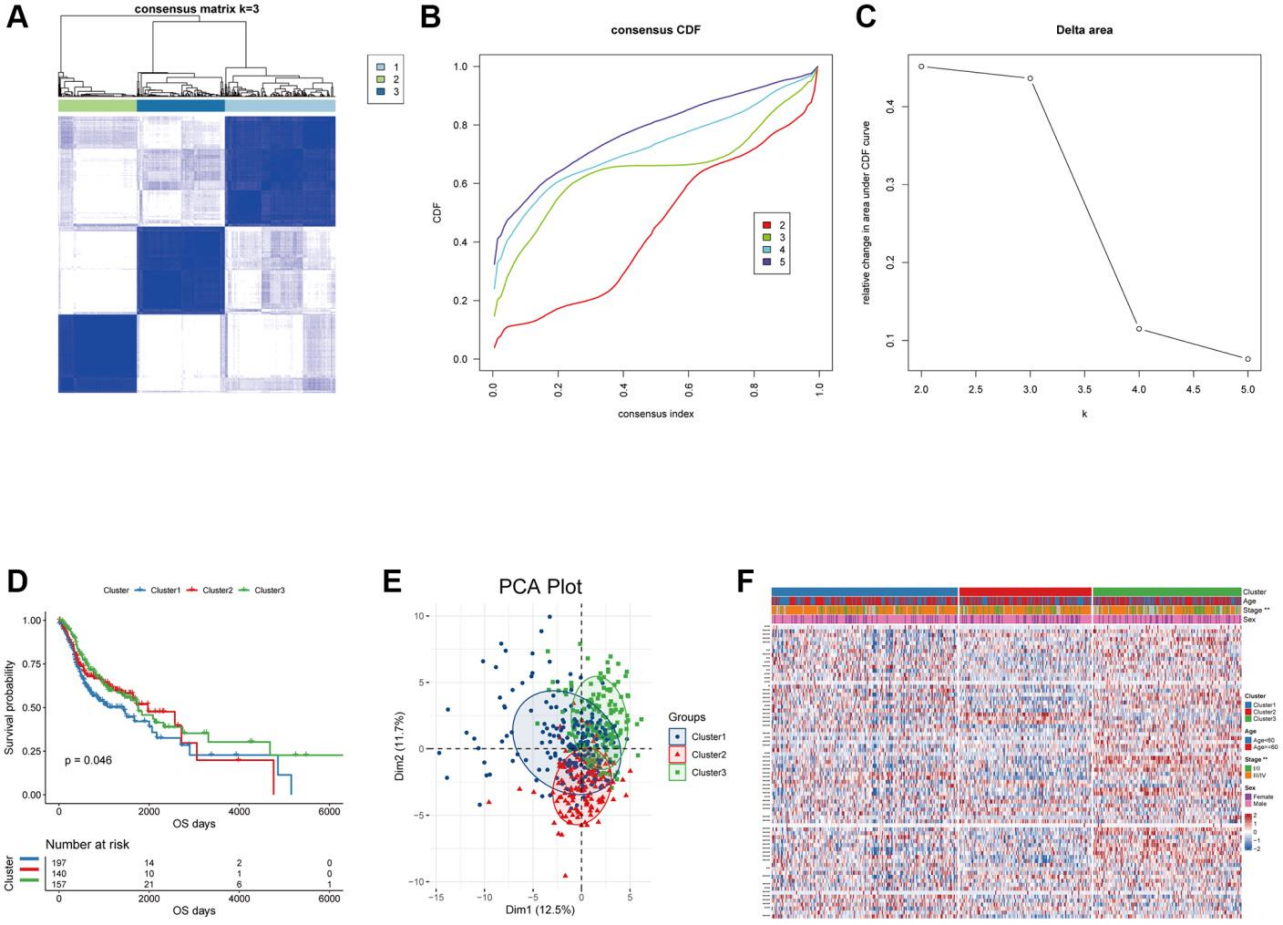
**Unsupervised clustering analysis for the head and neck squamous carcinoma samples based on the expression of oxidative stress-related genes.** (**A**) Consensus matrices of the TCGA-HNSC cohort with k = 3; 1, 2 and 3 denote the three subgroups. (**B**) The CDF plot of unsupervised clustering analysis. (**C**) Relative change in area under CDF curve for k = 2–5. (**D**) OS survival curves of the three oxidative stress-related subgroups. (**E**) Visualization of the results of the PCA analysis for the oxidative stress-related genes. (**F**) Heat map of the expression of the oxidative stress-related genes in the three subgroups.

The analysis flow chart was shown in (Supplementary Figure 1). Based on the expression of 74 oxidative stress-related genes in the TCGA-HNSCC dataset, consensus clustering analysis was performed using “ConsensusClusterPlus”. We finally identified three oxidative stress-related subgroups, termed Cluster1, Cluster2 and Cluster3 (*n* = 197/140/157, Figure 1A–1C). The three subgroups significantly differed in terms of prognosis, with Cluster1 having poorer OS (Figure 1D). Three distinct oxidative stress patterns were identified through analyses of the expression profile of oxidative stress-related genes (Figure 1E). Significant differences in tumor staging between distinct subgroups of patients were observed (*p* < 0.05, Figure 1F).

#### Construction of the oxidative stress-related prognostic signature

To evaluate the oxidative stress-related patterns of individual patients, we constructed an oxidative stress-related signature to predict the prognosis of HNSCC patients based on the DEGs between oxidative stress expression patterns.

We initially screened 216 DEGs among the three oxidative stress expression patterns using R package “limma” (Supplementary Figure 2A–2C). We then conducted GO and KEGG enrichment analyses for the DEGs using the “clusterProfiler” package. Genes were significantly enriched in biological processes including epidermal development, epidermal cell differentiation and humoral immunity (Supplementary Figure 2D–2G).

**Figure 2:**
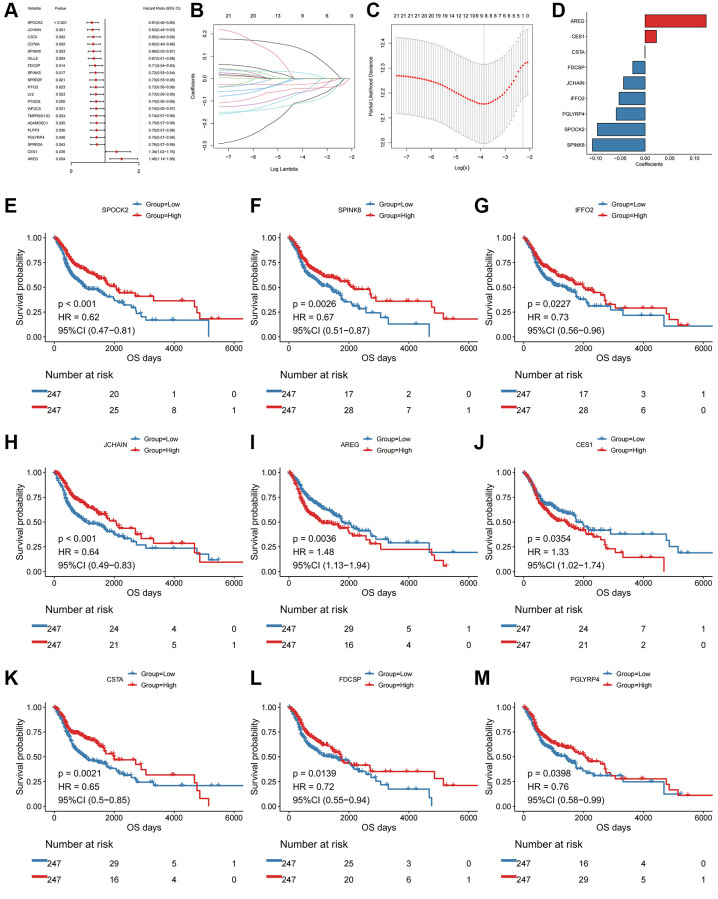
**Univariate Cox and LASSO regression analysis of the DEGs.** (**A**) Forest plot of the top 20 prognostic genes. (**B**) Confidence interval of each Lambda in the LASSO regression analysis. (**C**) Trajectory change of LASSO regression independent variables, the abscissa axis indicates the logarithm of the independent variable Lambda, and the vertical axis indicates the coefficient of the independent variable. (**D**) LASSO regression coefficient of key prognostic genes. The abscissa axis represents coefficients; The vertical axis represents different gene names. (**E**) Kaplan-Meier curve of SPOCK2 involved in the oxidative stress-related prognostic signatures. (**F**) Kaplan-Meier curve of SPINK6 involved in the oxidative stress-related prognostic signatures. (**G**) Kaplan-Meier curve of IFFO2 involved in the oxidative stress-related prognostic signatures. (**H**) Kaplan-Meier curve of JCHAIN involved in the oxidative stress-related prognostic signatures. (**I**) Kaplan-Meier curve of AREG involved in the oxidative stress-related prognostic signatures. (**J**) Kaplan-Meier curve of CES1 involved in the oxidative stress-related prognostic signatures. (**K**) Kaplan-Meier curve of CSTA involved in the oxidative stress-related prognostic signatures. (**L**) Kaplan-Meier curve of FDCSP involved in the oxidative stress-related prognostic signatures. (**M**) Kaplan-Meier curve of PGLYRP4 involved in the oxidative stress-related prognostic signatures.

We next performed univariate Cox regression analysis. In total, 22 of the DEGs were significantly associated with OS in the TCGA-HNSCC cohort, including SPOCK2, JCHAIN, CSTA, CD79A (Figure 2A, top20 genes were shown in order of hazard ratio from low to high).

Although identified genes with prognostic efficacy in HNSCC patients were identified by univariate Cox regression analysis and log-rank tests, redundant factors were removed to control the risk of overfitting and LASSO-Cox regression analysis was performed based on the 22 genes. A 10-fold cross-validation was performed under optimal conditions to determine the penalty parameter (λ) of the model. The nine most predictive factors affecting OS were screened out (Figure 2B, 2C). Among those, AREG and CES1 were retained as valid risk factors for prognosis, whilst CSTA, FDCSP, JCHAIN, IFFO2, PGLYRP4, SPOCK2 and SPINK6 were retained as protective prognostic factors (Figure 2D–2M). All factors constituted a prognostic risk model that was related to oxidative stress in HNSCC. Based on the expression levels of those genes and the linear combination of their corresponding weights, we assessed the prognostic risk score related to oxidative stress for each patient. The specific computational formula was listed as follows [14, 15]:

Score = -SPOCK2 × 0.096-JCHAIN × 0.044-CSTA × 0.0004-SPINK6 × 0.106 + AREG × 0.123-FDCSP × 0.025-IFFO2 × 0.053 + CES1 × 0.023-PGLYRP4 × 0.058.

**Figure 3:**
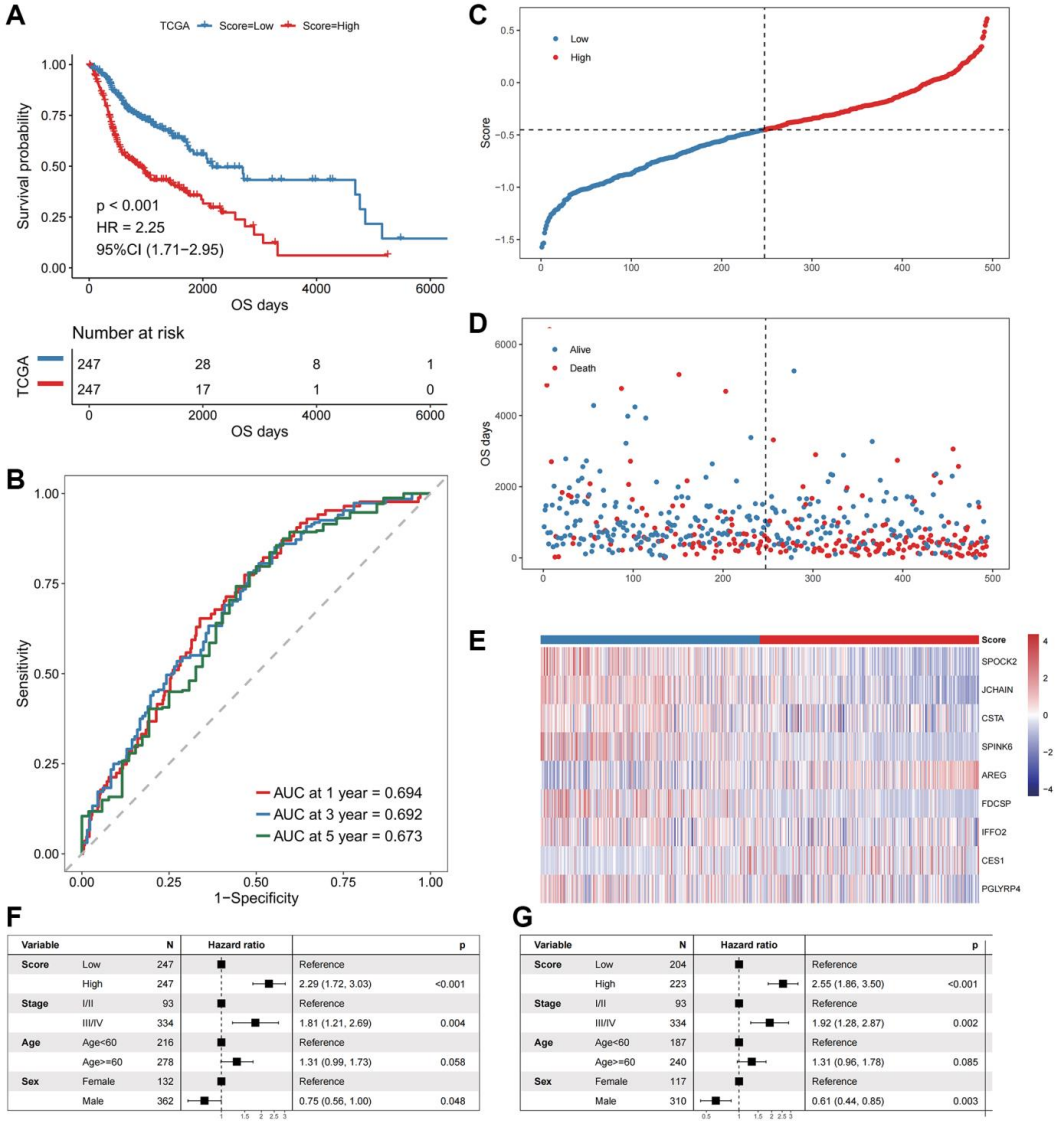
**The performance of the model in the training cohort.** (**A**) The survival curve of patients in high- and low-Score groups. The abscissa axis represents the overall survival days; The vertical axis represents survival probability; Different colors represent different subgroups. (**B**) The ROC curve for predicting the 1-, 3-, and 5-year survival of HNSCC patients according to the Score. The abscissa axis represents specificity; The vertical axis represents sensitivity; Different colors represent different time subgroups. (**C**) The distribution of the Score in HNSCC patients. The abscissa axis represents time; The vertical axis represents cumulative score; Different colors represent different score subgroups. (**D**) The survival status of HNSCC patients. The abscissa axis represents time; The vertical axis represents overall survival days; Different colors represent different survival status. (**E**) The expression profiles of the nine genes involved in the model of each sample, the Score increasing gradually from left to right. (**F**) Forest plots show the results of univariate Cox regression analyses performed on clinical characteristics. (**G**) Forest plots show the results of multivariate Cox regression analyses performed on clinical characteristics.

Based on the oxidative stress-related prognostic signature, we calculated the risk score of each patient in the TCGA-HNSCC cohort and divided patients into high- and low- risk groups according to the median values. Kaplan-Meier curve analysis and log-rank tests indicated that the OS of patients in the high-risk group were significantly shorter (log-rank *p*-value < 0.001, Figure 3A). The AUCs of the patients at 1, 3, and 5 years were 0.694, 0.692, and 0.673 (Figure 3B), respectively, indicating an accurate characterization of OS.

We next explored the independence of the prognostic signature in patients with HNSCC in TCGA (Figure 3C–3G). A multivariate Cox regression model was constructed jointly based on prognostic risk score and clinical characteristics. The results indicated that the prognostic risk score was an independent prognostic factor (HR = 2.55, *p*-value < 0.001, Figure 3G).

#### Validation of the prognostic signature in an independent dataset

**Figure 4:**
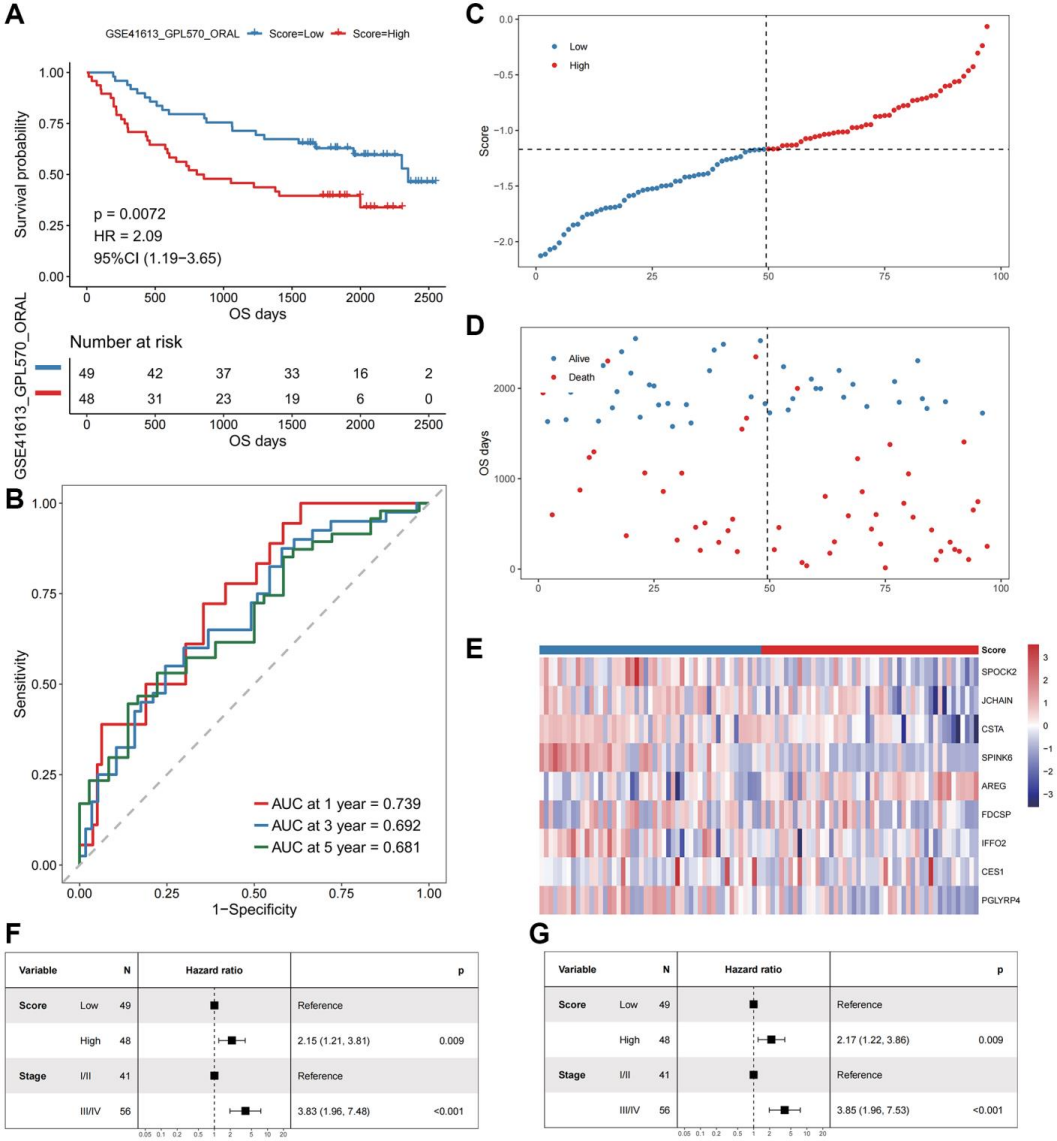
**The performance of the model in the validation cohort (GSE41613).** (**A**) The survival curve of patients in high- and low-Score groups. The abscissa axis represents the overall survival days; The vertical axis represents survival probability; Different colors represent different subgroups. (**B**) The ROC curve for predicting the 1-, 3-, and 5-year survival of HNSCC patients according to the Score. The abscissa axis represents specificity; The vertical axis represents sensitivity; Different colors represent different time subgroups. (**C**) The distribution of the Score in HNSCC patients. The abscissa axis represents time; The vertical axis represents cumulative score; Different colors represent different score subgroups. (**D**) The survival status of HNSCC patients. The abscissa axis represents time; The vertical axis represents overall survival days; Different colors represent different survival status. (**E**) The expression profiles of the nine genes involved in the model of each sample, the Score increasing gradually from left to right. (**F**) Forest plots show the results of univariate Cox regression analyses performed on clinical characteristics. (**G**) Forest plots show the results of multivariate Cox regression analyses performed on clinical characteristics.

To assess the robustness and generalizability of the oxidative stress-related prognostic signature, we adopted GSE41613 as an independent validation cohort. Patients were divided into high- and low-risk groups based on the signature. The OS of patients in the high-risk group was significantly shorter than the low-risk group (Figure 4A). The AUCs of the patients at 1, 3, and 5 years were 0.739, 0.692, and 0.681, respectively (Figure 4B). A multivariate Cox regression model was constructed based on the prognostic risk score and clinical characteristics. The results were consistent with the training cohort, which validated the risk score as an independent prognostic factor (Figure 4C–4G).

### Oxidative stress expression patterns resolved by single-cell transcriptomics

To further investigate the role of oxidative stress in HNSCC at the single-cell level, published single-cell sequencing datasets of HNSCC patients were analyzed in the GEO database (GSE103322). Based on the 2205 malignant cells extracted, two malignant subgroups were identified, termed CellType 0 and CellType 1 (Supplementary Figure 3A). CellType 0 contained 1328 cells and CellType 1 contained 877 cells. Malignant cells were divided into CellGroups according to the median CellScore. The results indicated that the proportion of cells with high CellScores exceeded that of CellType 1. A higher number of low CellScores were observed in CellType 0 (Supplementary Figure 3B–3D).

To explore the biological significance of the prognostic signature in tumor cells, we performed trajectory analysis based on 2215 malignant cells. Three differentiation states were observed (Supplementary Figure 3E). To determine the starting point of the trajectory and pseudotime analysis, the tumor cell stemness index was used to identify the starting point. The results showed that cells with a high stemness index were mainly distributed into differentiation state 3 (Supplementary Figure 3G), consistent with the trend of the trajectory plot of pseudotime analysis in (Supplementary Figure 3F). Differentiation state 3 was therefore established as the starting point. The stemness of tumor cells gradually decreased along the pseudotime sequence. CellType 1 cells that transformed to CellType 0 were shown in (Supplementary Figure 3H). Cells with high scores are mainly distributed to differentiation state 3, corresponding to cells with high stemness (Supplementary Figure 3I, 3J).

### Differential activation of transcription factors between high and low CellScore groups related to OSPS

We next investigated the activation of transcription factors in high and low CellScore groups. Seven regulon modules (M1~M7) were identified according to the linkage specificity index between different transcription factors (Supplementary Figure 4A). The average activity of M3 in the CellScore high group exceeded that of the low group. In contrast, the average activity of M7 in the CellScore high group was lower than that of the low group (Supplementary Figure 4B). This reflected differences in activated TF in different malignant cell subgroups. Through mapping the activity of the transcription factors to UMAP (Uniform Manifold Approximation and Projection) and trajectory analysis, we observed significant differences in the distribution of M3 and M7 mean activity in different subgroups in addition to differentiation states (Supplementary Figure 4D, 4E). We calculated RSS (Regulon Specificity Score) for each regulon in CellScore high- and low-groups, which were ranked from high to low. The rank of regulons in each CellGroup and the distribution of RSS are shown in Supplementary Figure 4C. The regulon with high RSS correlated with cell specificity (Supplementary Figure 4C). The RSS distribution of the top 2 regulons in the CellScore high group of malignant cells is shown in Supplementary Figure 4F, 4G. The RSS distribution of the top 2 regulons in the CellScore low group is shown in Supplementary Figure 4H, 4I. The RSS distribution of the remaining regulons in the Low group are shown in Supplementary Figure 2. The functional enrichment results of each regulon are shown in Supplementary Figure 3.

### Relationship between OSPS and the tumor microenvironment

**Figure 5:**
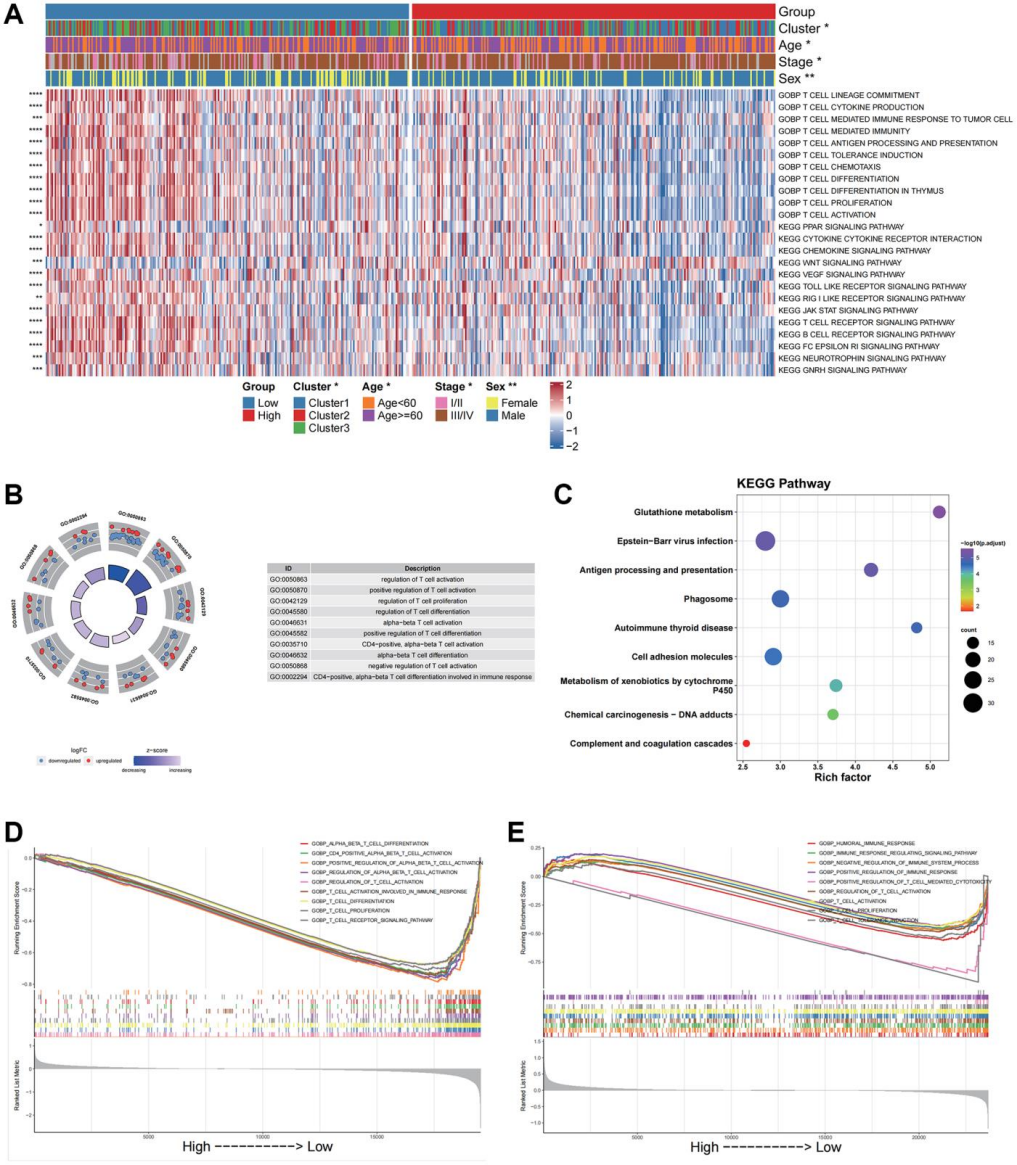
**Functional enrichment analysis for different groups in bulk data and scRNA data.** (**A**) GOBP and KEGG pathway enrichment analysis of the bulk data calculated by GSVA. (**B**) GO enrichment analysis of the scRNA data by the “clusterProfiler”. (**C**) KEGG pathway enrichment analysis of the scRNA data by the “clusterProfiler”. The left column represents the name of the enrichment pathway, the balloon in the middle column represents the weight of the corresponding pathway, and the right column represents the corresponding annotation. (**D**) GSEA analysis results based on the bulk data. The abscissa axis represents the high and low grouping; The vertical axis represents the Running Enrichment Score. Curves of different colors represent different pathways. (**E**) GSEA analysis based on the scRNA data. The abscissa axis represents the high and low grouping; The vertical axis represents the Running Enrichment Score. Curves of different colors represent different pathways.

Based on the bulk RNA-seq data, we calculated both pathway and biological processes through KEGG and GOBP analyses using the “GSVA” algorithm. Differences in activities between subgroups were compared through rank sum tests. The results indicated significant differences in T cell differentiation involving immune responses, T cell-mediated cytotoxicity and cytokine-cytokine receptor interactions (Figure 5A).

Based on the scRNA data, we then analyzed differentially characterized genes between high and low CellGroups and performed GO and KEGG enrichment analyses using “clusterProfiler”. The results indicated that the genes were significantly enriched in immune-related biological processes such as the regulation of T cell activation, and KEGG pathways including phagosome and antigen processing and presentation (Figure 5B, 5C). These results were consistent with those of the bulk dataset.

GSEA analysis was performed to investigate differences in biological processes between high- and low score groups for both bulk and scRNA data. The results indicated that the low-risk group was significantly enriched in immune-related pathways including T-cell activation in both the bulk and scRNA datasets (Figure 5D, 5E).

Based on the bulk RNA-seq data, the percentage of immune cell infiltration in each tumor samples were estimated. The OSRS was found to be negatively correlated with immune and stromal score but positively correlated with tumor purity (Supplementary Figure 5A–5D). Significant differences in the infiltration of T cells CD8, T cells CD4 memory activated, T helper cells, naïve B cells, dendritic cells and NK cells between different OSRS groups were observed (Supplementary Figure 5E).

### Differences in cellular communication between high and low Score groups of OSPS

We performed cellular communication analyses between immune and tumor cells using the “CellChat” package. Extensive cellular communications amongst all cell subgroups were observed (Supplementary Figure 6A, 6B). When both incoming and outgoing signals were distinguished, malignant tumor cells and dendritic cells were outgoing signaling cells, whilst T cells, B cells, mast cells and macrophages were incoming signal receivers (Supplementary Figure 6C).

Using the relationship between Cophenetic and pattern number, we identified 2 patterns of cell subgroups, in which high neoplastic and low neoplastic belonged to Pattern 2, with corresponding pathways including LAMININ, MK and other pathways related to tumor malignant progression (Supplementary Figure 6D, 6E). T cells, B cells, Mast cells, macrophages and dendritic cells belonged to Pattern 1, which included immune-related pathways such as MHC-I (Supplementary Figure 6F).

### Value of the OSPS in predicting immunotherapy efficacy and drug sensitivity

We further explored the predictive value of the OSPS in the prognosis of patients in the immunotherapy cohort PRJEB23709. We found that patients with a low OSRS had an improved prognosis (Supplementary Figure 7A). Patients in the immunotherapy response group had significantly lower OSRS scores than the non-responder group (Supplementary Figure 7B). Significant differences in the proportion of patients responding or not responding to immunotherapy amongst the high- and low- score groups were observed (*p* < 0.05), with more than 75% of patients in the low score group responding to immunotherapy (Supplementary Figure 7C). These results suggested that the low score group is likely to benefit from immunotherapy.

The IC50 values of drugs in the training cohort were predicted using R package “oncoPredict” and drug information in GDSC and CTRP databases combined with the expression profile of the training cohort. We compared “Spearman” correlation values between the OSRS and log2(IC50) values of each drug. Drugs were ranked according to the absolute value of correlation coefficient from the largest to the smallest. We selected the top 6 drugs with the most significant positive and negative correlation, respectively (Supplementary Figure 7D, 7F; *p* < 0.05). Significant differences in drug log2(IC50) were observed between high- and low- score groups (Supplementary Figure 7E–7G). CTRP results are shown in (Supplementary Figure 4).

### Prognostic significance of SPINK6 expression in nasopharyngeal squamous cell carcinoma

**Figure 6:**
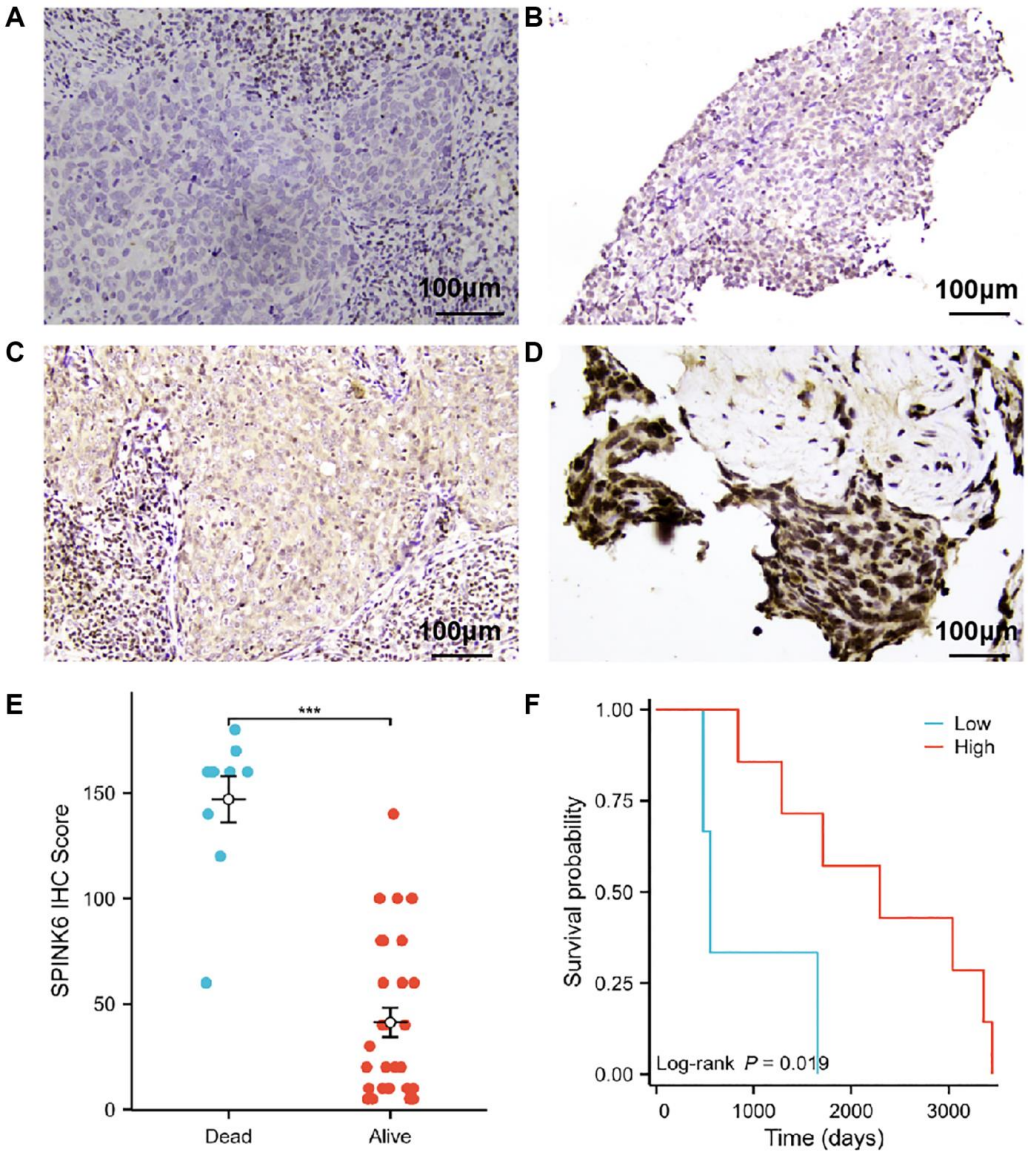
**Prognostic significance of SPINK6 protein expression in nasopharyngeal squamous cell carcinoma.** (**A**–**D**) Representative photomicrographs of SPINK6 protein expression by IHC magnified 200 times under a light microscope. (**A**) Staining intensity of SPINK6 protein was negative (0). (**B**) Staining intensity of SPINK6 protein was weak (1+). (**C**) Staining intensity of SPINK6 protein was moderate (2+). (**D**) Staining intensity of SPINK6 protein was strong (3+). (**E**) Comparison of high and low SPINK6 IHC Score groups in validation cohort. (**F**) KM survival curves of high and low SPINK6 IHC Score groups in validation cohort.

IHC was performed on clinical pathological sections of 41 patients. The staining intensity of SPINK6 in tumor cells was scored negative (0), weak (1+), moderate (2+) or strong (3+) (Figure 6A–6D). SPINK6 expression analysis was performed using the IHC score (ranging from 0 to 300), which involved multiplying the percentage of positive cells by the staining intensity. Using the median SPINK6 IHC Score as the cut-off, patients were divided into the high and low SPINK6 expression groups. A total of 20 patients had an IHC score ≥60. Ten patients died and were used for final survival analysis. Survival curves indicated that patients with low SPINK6 IHC scores had poorer survival (log-rank *p* = 0.019; Figure 6E, 6F).

### Validating the expression of the genes involved in the prognostic signature through IHC staining results

To further prove the reliability of the results in this study, we validated the protein expression levels of the genes involved in the prognostic signature using the IHC staining images in the Human Protein Atlas (HPA) database (https://www.proteinatlas.org/). First, we compared the RNA expression levels of the genes between normal and tumor tissues in TCGA by GEPIA 2 database (http://gepia2.cancer-pku.cn/). Then the IHC staining images of three genes that showed significantly differential expression in TCGA were obtained. Compared with normal head and neck tissues such as thyroid gland, nasopharynx, oral tissue or salivary gland, the protein expression levels of CES1, CSTA and FDCSP genes were decreased in HNSCC (Supplementary Figure 8B–8F, 8H–8J, 8L, 8M), which is consistent with the RNA expression levels in TCGA (Supplementary Figure 8A, 8G and 8K).

## DISCUSSION

Studies had demonstrated that oxidative stress was aberrant in HNSCC [9–11, 17–20]. The relationships between oxidative stress and the patients' prognosis, TME, responses to immunotherapy and drugs were however poorly understood.

In this study, we performed consensus clustering analysis based on the expression profiles of genes related to oxidative stress. Patients were classified into three distinct oxidative stress expression patterns with varying prognosis (Figure 1). To further explore the mechanisms leading to the differences in prognosis, we identified DEGs between the three oxidative stress patterns and performed KEGG and GO enrichment analyses (Supplementary Figure 2). The results indicated the DEGs were enriched in antimicrobial humoral immune responses (Supplementary Figure 2E) [21].

We then performed a prognostic analysis for the DEGs. An oxidative stress-related gene scoring model was designed through univariate and multivariate Cox regression analyses, identifying a prognostic signature that could predict the patients' survival (Figure 2). This signature was robust and independently predictive in both training and validation cohorts (Figures 3, 4). The prognostic signature was composed of nine genes, including AREG and CES1 identified as risk factors, and CSTA, FDCSP, JCHAIN, IFFO2, PGLYRP4, SPOCK2 and SPINK6 identified as protective factors (Figures 2E and 6). AREG is a ligand of EGFR, which is usually overexpressed in tumors and associated with poor prognosis and resistance to chemo- and radiotherapy [22]. It had also been reported to be an adverse factor for the prognosis of patients with HNSCC [22–25]. High expression of AREG was also related to chemotherapy resistance in HNSCC [26, 27]. Low CSTA expression could promote lymphatic metastasis and was associated with poor OS in HNSCC patients [28, 29]. FDCSP expression was reported to be related to TP53 mutational status and chemokine pathways. High expression of this gene was favorable for the prognosis of patients with HPV+ HNSCC [30]. JCHAIN, PGLYRP4 and SPINK6 had been reported as protective factors for HNSCC patients, consistent with the results in this study [31–33].

For CES1, IFFO2 and SPOCK2, reported in other diseases [34–36], were rarely brought to notice in HNSCC. In this study, CES1 was a risk factor, whilst IFFO2 and SPOCK2 were protective factors for HNSCC patients. These three genes might therefore represent prognostic markers for HNSCC and the oxidative stress-related signature composed of nine genes might represent a novel potential tool to predict the HNSCC patients' survival.

Given that single-cell sequencing was an advanced and robust method for TME information in HNSCC, we further analyzed the scRNA-seq data [37–39]. We identified two subgroups of malignant cells and calculated the oxidative stress-related CellScores based on the expression of the nine gene prognostic signature (Supplementary Figure 3A–3D). We then performed trajectory and pseudotime analyses using the stemness index of tumor cells as the starting point (Supplementary Figure 3E–3J). The results suggested that a high CellScore was associated with a high stemness index. Cancer stem cells (CSCs) played a critical role in the initiation, relapse, metastasis, and chemoresistance of multiple types of cancers including HNSCC [40, 41]. Malignant cells with high CellScores might be more difficult to target through anti-humoral immune responses and treatment. Patients with high oxidative stress-related scores had a poorer prognosis. Significant differences in the activation of transcription factors between CellScore high- and low groups were also observed (Supplementary Figure 4).

SP3 and ATF2 were the most highly expressed transcription factor in the CellScore high group. TP53 and CD59 were most highly expressed in the CellScore low group (Supplementary Figure 4F–4I). SP3 was a member of SP-family and was an oncogene that played a pivotal role in cell proliferation and metastasis of various tumors [42–46]. ATF2 was also an oncogene that is associated with the progression and resistance to anti-tumor therapy, including HNSCC [47–51]. TP53 was a tumor suppressor that was frequently mutated and inactivated in HNSCC [17, 52–55]. CD59 could inhibit complement and CD8+ T cell activation, leading to immune evasion and immune checkpoint blockade [56], which was also overexpressed in HNSCC and regulated tumor metastasis and prognosis [57–59]. We speculated that the high expression of pro-humoral transcription factors and the low expression of anti-humoral TP53 results in a poorer prognosis of HNSCC patients with high scores.

The temporal and spatial heterogeneity of tumor samples were common [60, 61], which affected the detection of tumor biomarkers in varying degrees. By comparing the results of mixed sample (TCGA) and single cell sequencing analyses, it was found that our analysis results had a certain degree of universality and were weakly affected by the heterogeneity of tumor samples, which would facilitate clinical applications (Figures 1–4 and Supplementary Figures 3, 4). This universality might also increase the potential for clinical conversion of our research results.

We subsequently investigated the relationship between oxidative stress-related prognostic signatures and the TME. The results indicated that biological processes or pathways related to anti-tumoral immune responses were significantly activated in the low score group, including T cell activation, T cell mediated immunity and T cell receptor signaling. Some pro-humoral pathways such as WNT signaling were inactivated in the low Score group (Figure 5A–5C). GSEA analysis showed that the DEGs between the high- and low- score groups were enriched in immune activation associated biological processes in both bulk RNA and scRNA datasets (Figure 5D, 5E). Following the analysis of immune cell infiltration in the bulk datasets, we found that the oxidative stress-related score negatively correlated with the ImmuneScore, T cells CD8, T cell CD4 memory activated cells and M1 macrophages (Supplementary Figure 5), confirming that a low OSRS was related to anti-humoral immune activation, as CD8+ T cells, CD4+ T cells and M1 macrophages played essential roles in tumor suppression in the TME [62–64]. Further analyses showed significant differences in cellular communication between high- and low- OSRS groups (Figure 5 and Supplementary Figure 6). We therefore speculated that the activation of anti-humoral responses in patients with low OSRS might be an important indicator of prognosis.

Immunotherapy by immune checkpoint inhibitors (ICIs) was a major breakthrough in cancer treatment. Its efficacy was however variable and limited to subsets of patients [65]. The TME landscape including the infiltration of CD8+ T cells, CD4+ T cells and macrophages was closely related to ICI immunotherapy [63, 66, 67]. Those cells displayed significant differences in low- and high score groups, with the low score group showing superior clinical outcomes to immunotherapy (Supplementary Figure 7A–7C). The efficacy of some anti-cancer agents also differed between high- and low- score groups of tumor cells (Supplementary Figure 7D–7G). These results demonstrated how the oxidative stress-related gene scoring model held value for prediction of the clinical outcomes of immunotherapy in HNSCC patients. The subsequent immunohistochemical results further verified the authenticity of the genes included in the scoring prediction model (Figure 6 and Supplementary Figure 8). Consistency between online data analysis results and clinical validation results further suggested that the predictive model might have a better clinical conversion potential.

Though plenty of work had been made, the lack of specific functional validation of the 9 signature genes was still the major limitation of the study. Therefore, the conclusion on the predictive performance of this prediction model was only based on the current analysis results and partially validations. The conclusion still had some degree of hypothesis. In the future, it is still necessary for us to conduct a large number of basic experiments and clinical cohort studies to further verify the above conclusions.

In summary, we constructed an oxidative stress-related prognostic signature to evaluate the oxidative stress patterns of individual patients. Based on the signature, patients were divided into high- and low- risk groups. The prognosis of the high score patients was poor. Univariate and multivariate Cox analysis indicated that the oxidative stress-related signature was an independent prognostic factor, which was confirmed in an independent validation cohort and clinical samples. Immunotherapy data confirmed that our prognostic signature also held predictive value for the clinical outcomes of immunotherapy.

## CONCLUSIONS

As an independent prognostic factor, the described risk prediction model based on oxidative stress genes showed good predictive value for the prognosis of HNSCC and immunotherapeutic responses.

## Supplementary Materials

Supplementary Figures
