# Supplementary material for: A novel oxidative stress-related gene signature as an indicator of prognosis and immunotherapy responses in HNSCC
Source: Aging (Albany NY). 2023 Dec 28;15(24):14957–84. doi: 10.18632/aging.205323 (PMC10781479; doi:10.18632/aging.205323)
Supplement: Supplementary Figures [file aging-15-205323-s001.pdf]

SUPPLEMENTARY FIGURES

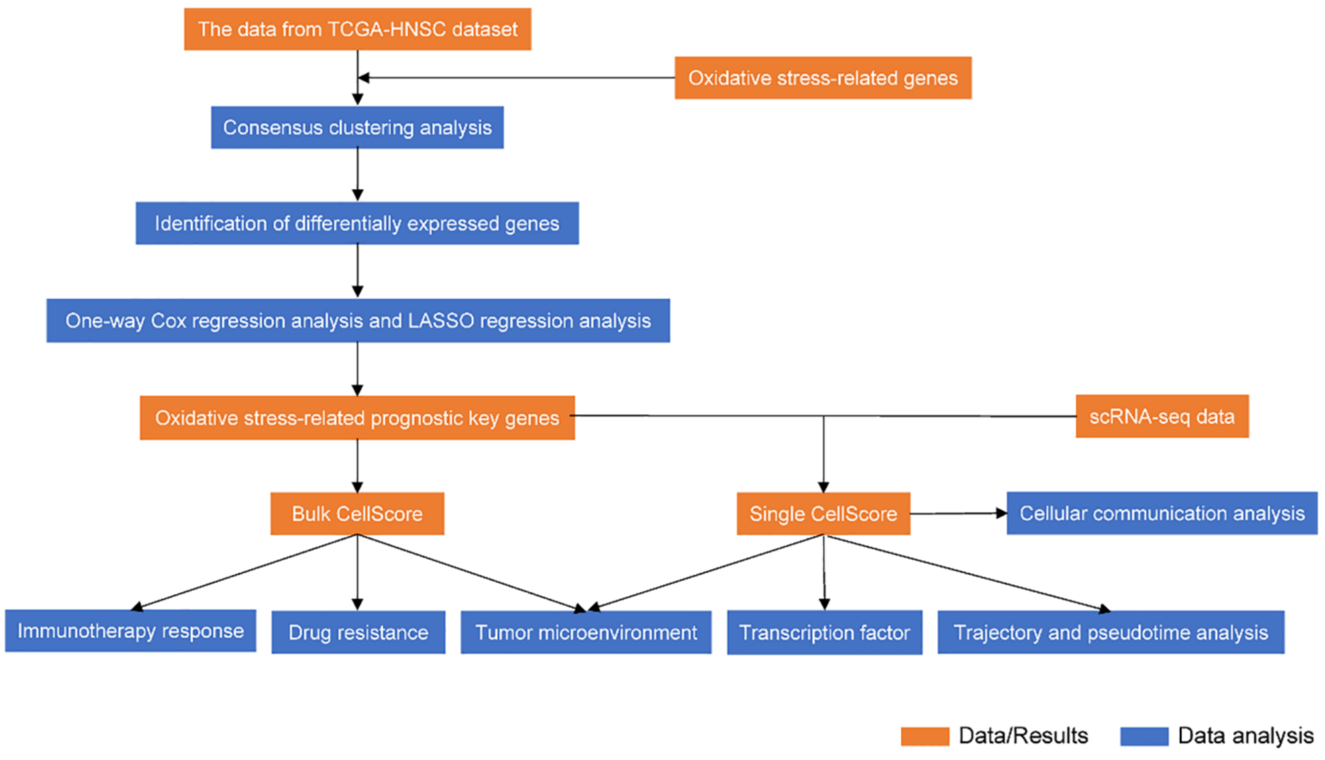

Supplementary Figure 1. The analysis flow chart of the study.

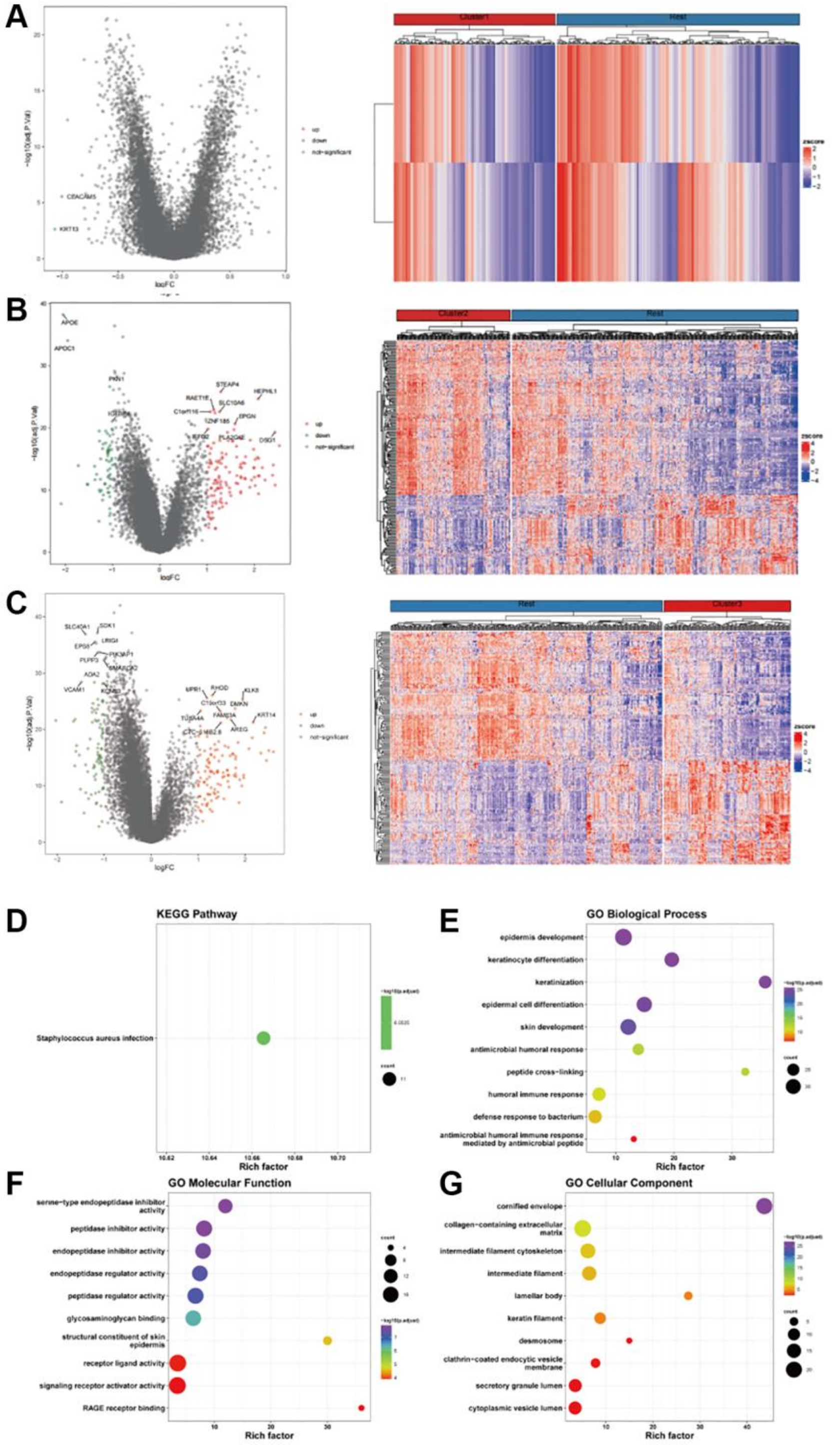

Supplementary Figure 2. Molecular differences between oxidative stress expression patterns. (**A**–**C**) The volcano plots and heat maps for the DEGs. (**D**) KEGG functional enrichment analysis for the DEGs. (**E**) GOBP functional enrichment analysis for the DEGs. (**F**) GOMF functional enrichment analysis for the DEGs. (**G**) GOCC functional enrichment analysis for the DEGs.

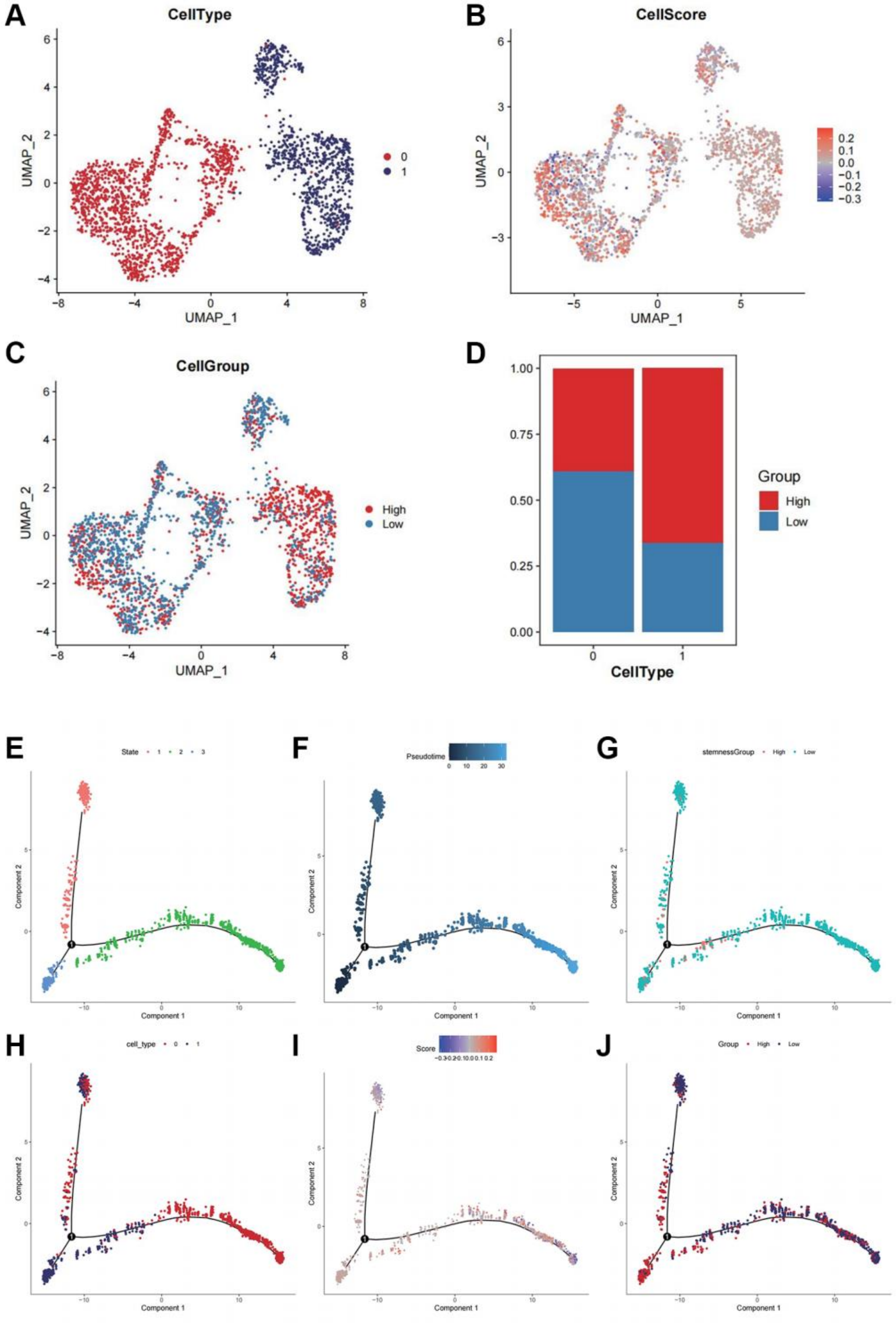

Supplementary Figure 3. Identification of malignant epithelial cell subgroups. (**A**) Distribution of malignant cell subgroups constructed by Uniform Manifold Approximation and Projection (UMAP); Different colors represent different cell types. A point within each group represents a cell. (**B**) CellScore distribution of malignant epithelial cells constructed by UMAP; Different colors represent different CellScores. (**C**) CellGroup distribution of malignant epithelial cells constructed by UMAP; Different colors represent different CellGroups. (**D**) The ratios of cells with high and low CellScore in different CellTypes. (**E**) The results of trajectory analysis for the tumor malignant cells based on different cell states; Different colors represent different states. (**F**) The results of trajectory analysis for the tumor malignant cells based on different pseudotime; Different colors represent different pseudotime. (**G**) The results of trajectory analysis for the tumor malignant cells based on different cell stemness groups; Different colors represent different cell stemness groups. (**H**) The results of trajectory analysis for the tumor malignant cells based on different cell types; Different colors represent different cell types. (**I**) CellScore; The results of trajectory analysis for the tumor malignant cells based on different cell scores; Different colors represent different cell scores. (**J**) The results of trajectory analysis for the tumor malignant cells based on different cell groups; Different colors represent different cell groups.

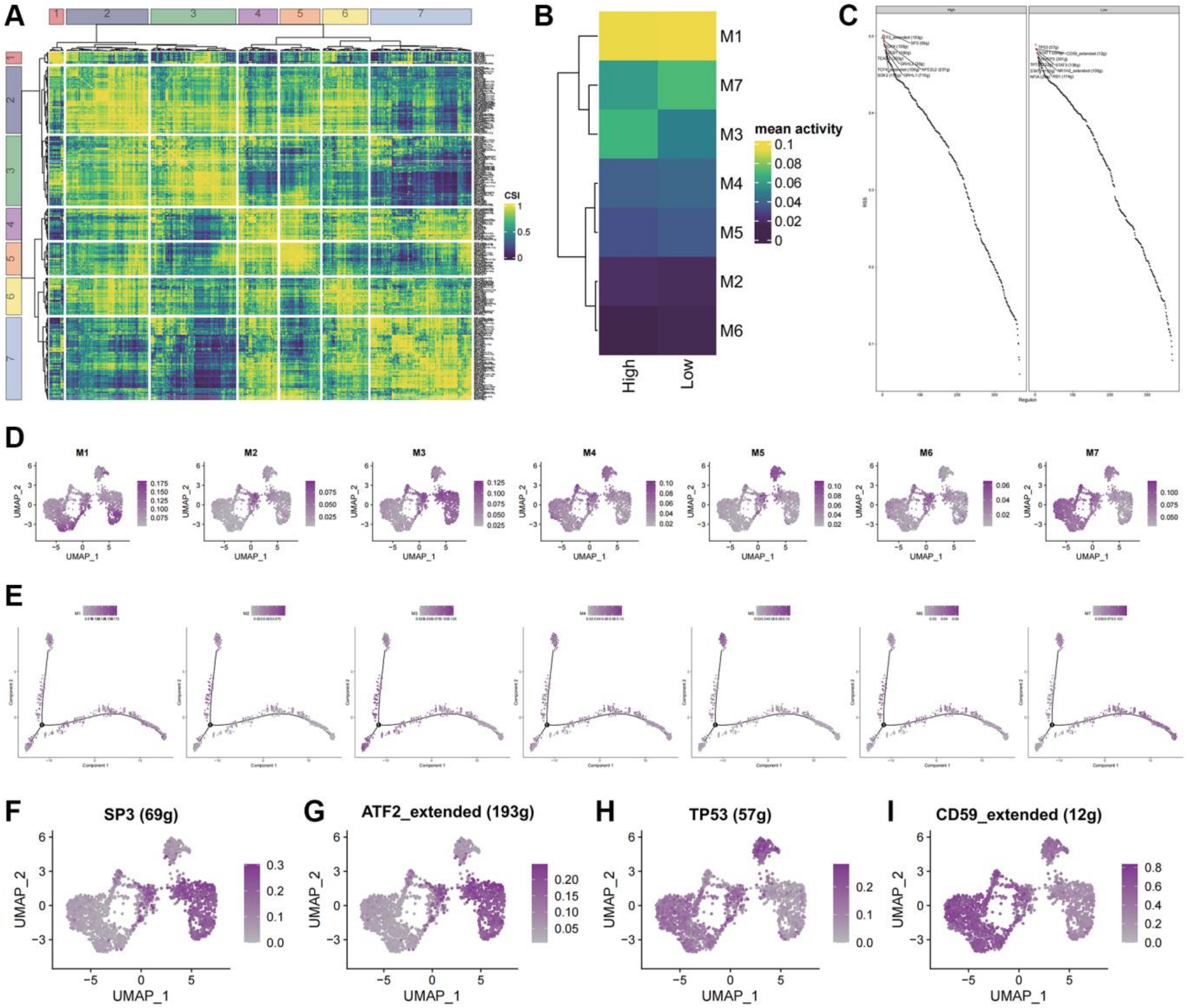

Supplementary Figure 4. Transcription factor regulatory network. (**A**) Transcription factors were divided into 7 regulon modules according to the synergistic effect of different transcription factors. (**B**) The association between CellGroup and regulon modules. (**C**) The distribution of each regulon's rank and RSS in CellScore high and low groups. (**D**) The distribution of mean activity of the regulon modules (M1-M7) constructed by UMAP. (**E**) The distribution of mean activity of the regulon modules (M1-M7) constructed by trajectory analysis. (**F**) The RSS distribution of the top 2 regulons (SP3) in CellScore high group constructed by UMAP. (**G**) The RSS distribution of the top 2 regulons (ATF2) in CellScore high group constructed by UMAP. (**H**) The RSS distribution of the top 2 regulons (TP53) in CellScore low group constructed by UMAP. (**I**) The RSS distribution of the top 2 regulons (CD59) in CellScore low group constructed by UMAP.

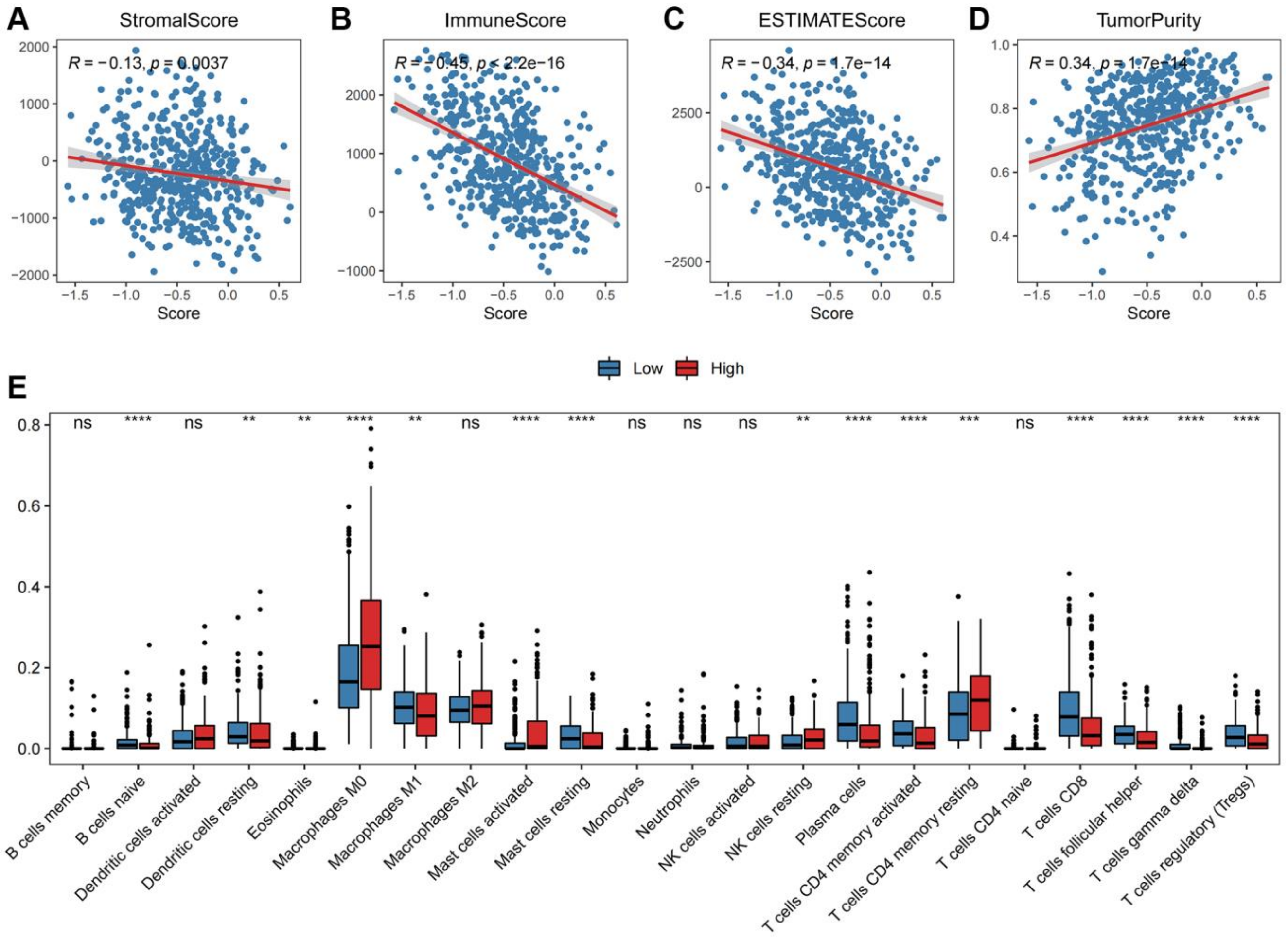

Supplementary Figure 5. Immune landscape of different subgroups based on bulk data. (**A**) Correlation analysis results between the proportion of immune infiltration and stromal score. (**B**) Correlation analysis results between the proportion of immune infiltration and ImmuneScore. (**C**) Correlation analysis results between the proportion of immune infiltration and ESTIMATEScore. (**D**) Correlation analysis results between the proportion of immune infiltration and tumor purity. (**E**) The fraction of various immune cell infiltration in high and low OSRS. The abscissa axis represents different immune cell names; The vertical axis represents the infiltration ratio; Different colors represent different subgroups.

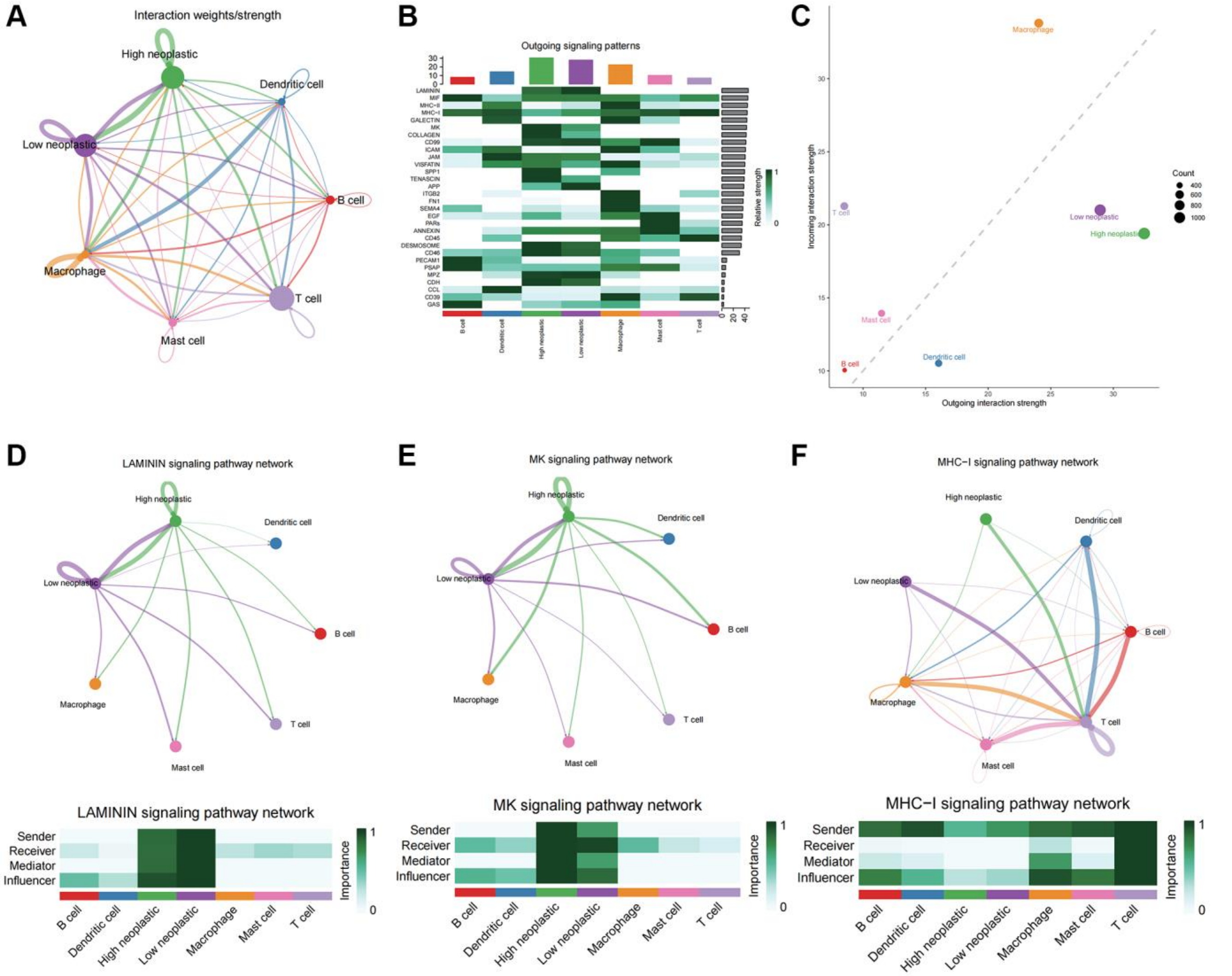

Supplementary Figure 6. Results of the cellular communication analysis. (**A**) The graph of all the cell–cell communications and cell–cell interaction network. (**B**) The statistical heat map of the signaling dominant of top 30 significant pathways. (**C**) The statistical point map of the signaling dominant. The abscissa axis represents outgoing interaction strength; The vertical axis represents incoming interaction strength; Different colors represent different immune cells. (**D**) The graph of the cell–cell communications and cell–cell interaction network of LAMININ signaling pathway. (**E**) The graph of the cell–cell communications and cell–cell interaction network of MK signaling pathway. (**F**) The graph of the cell–cell communications and cell–cell interaction network of MHC-I signaling pathway.

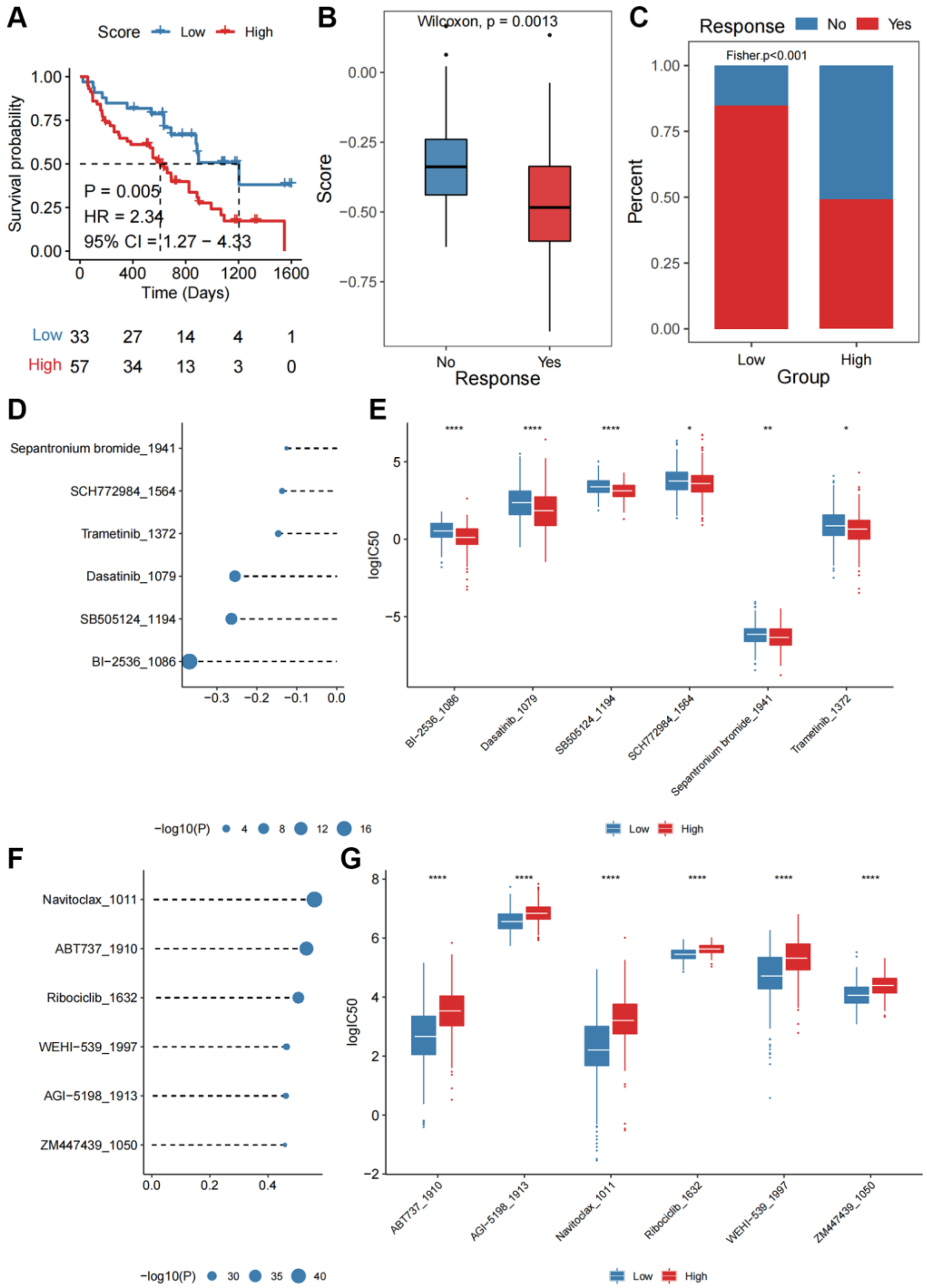

Supplementary Figure 7. The oxidative stress-related prognostic signature predicts the therapeutic outcomes of patients with HNSCC. (**A**) KM survival curves of high and low Score groups in PRJEB23709 cohort. The abscissa axis represents the survival time; The vertical axis represents the survival probability; Different colors represent different risk subgroups. (**B**) The OSRS distribution of different response groups in PRJEB23709 cohort. The abscissa axis represents different treatment responses; The vertical axis represents the risk score; Different colors represent different subgroups. (**C**) The percent of patients with response or no response to immunotherapy in the low Score and high Score groups. The abscissa axis represents different score groupings (low or high); The vertical axis represents the percentage; Different colors represent different subgroups of immunotherapy responses. (**D**) The correlation between the top six negatively correlated drugs and the Score. The abscissa axis represents the value of the correlation coefficient; The vertical represents six different drugs. (**E**) The logIC50 value of the top six negatively correlated drugs in low and high Score groups. The abscissa axis represents six different drug names; The vertical axis represents the logIC50 value; Different colors represent different risk score subgroups. (**F**) The correlation between the top six positively correlated drugs and the Score; The abscissa axis represents the value of the correlation coefficient; The vertical represents six different drugs. (**G**) The logIC50 value of the top six positively correlated drugs in low and high Score groups. The abscissa axis represents six different drug names; The vertical axis represents the logIC50 value; Different colors represent different risk score subgroups.

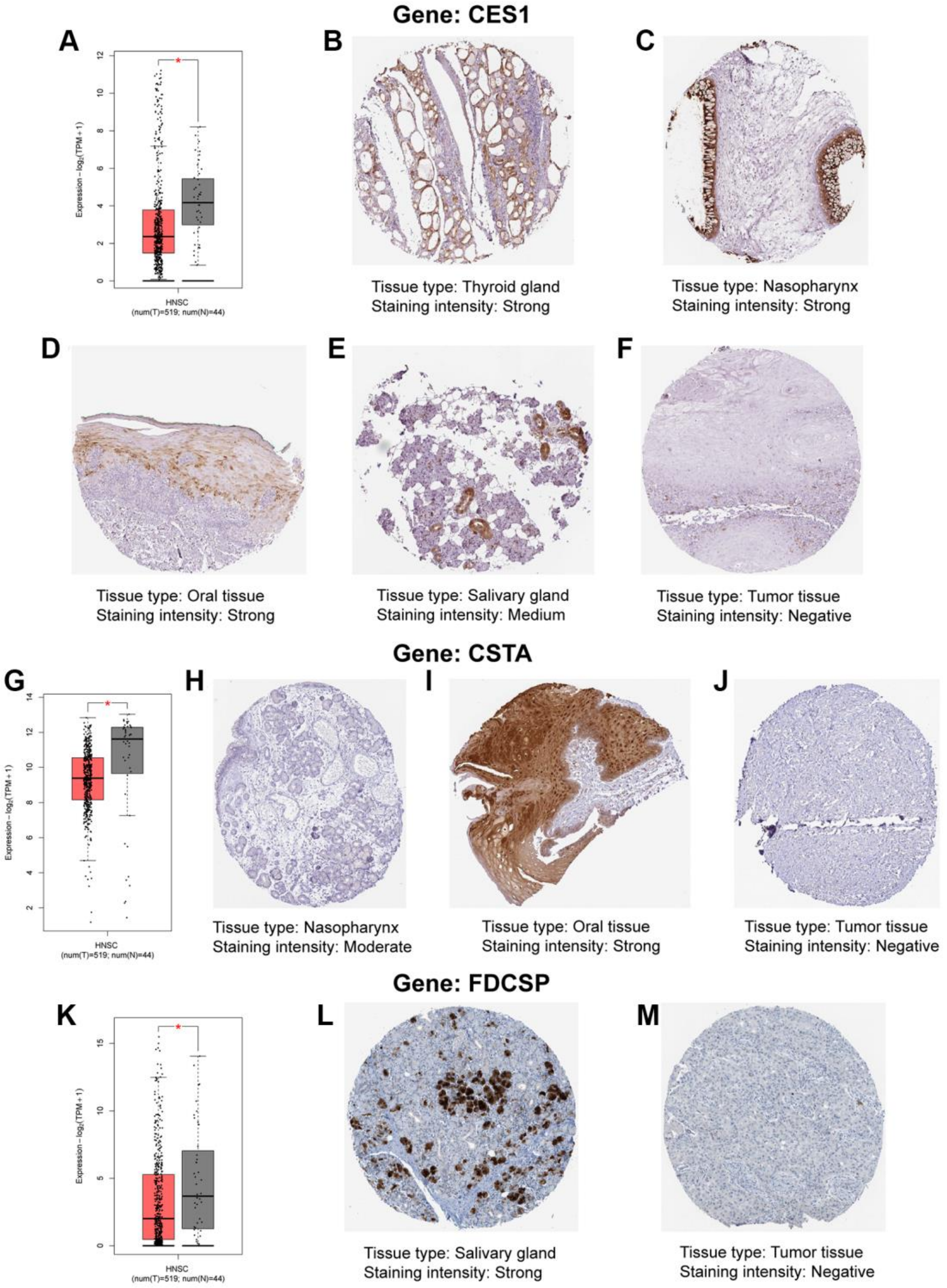

Supplementary Figure 8. IHC staining images of the genes involved in the prognostic signature. (**A**) The RNA expression level of CES1 in normal head and neck and HNSCC tumor tissues. (**B**–**F**) The IHC images showing the protein expression levels of CES1 in thyroid gland (**B**), nasopharynx (**C**), oral tissue (**D**), salivary gland (**E**) and HNSCC tumor tissue (**F**). (**G**) The RNA expression level of CSTA in normal head and neck and HNSCC tumor tissues. (**H**–**J**) The IHC images showing the protein expression levels of CSTA in nasopharynx (**H**), oral tissue (**I**) and HNSCC tumor tissue (**J**). (**K**) The RNA expression level of FDCSP in normal head and neck and HNSCC tumor tissues. (**L**, **M**) The IHC images showing the protein expression levels of FDCSP in salivary gland (**L**) and HNSCC tumor tissue (**M**).
